# Axillary artery injury combined with delayed brachial plexus palsy due to compressive hematoma in a young patient: a case report

**DOI:** 10.1186/1749-7221-3-9

**Published:** 2008-03-28

**Authors:** Keiichi Murata, Manabu Maeda, Atsushi Yoshida, Hiroshi Yajima, Kazuo Okuchi

**Affiliations:** 1 Departments of Orhopaedic Surgery, Nara Medical University, Kashihara, Japan; 2 Departments of Emergency and Critical Care Medicine, Nara Medical University, Kashihara, Japan

## Abstract

**Introduction:**

Axillary artery injury in the shoulder region following blunt trauma without association with either shoulder dislocation or fracture of the humeral neck has been previously reported. Axillary artery injury might also be accompanied with brachial plexus injury. However, delayed onset of brachial plexus palsy caused by a compressive hematoma associated with axillary injury after blunt trauma in the shoulder region has been rarely reported. In previous reports, this condition only occurred in old patients with sclerotic vessels. We present a case of a young patient who suffered axillary artery injury associated with brachial plexus palsy that occurred tardily due to compressive hematoma after blunt trauma in the shoulder region without association of either shoulder dislocation or humeral neck fracture.

**Case presentation:**

A 16-year-old male injured his right shoulder in a motorbike accident. On initial physical evaluation, the pulses on the radial and ulnar arteries in the affected arm were palpable. Paralysis developed later from 2 days after the injury. Functions in the right arm became significantly impaired. Angiography showed complete occlusion of the axillary artery. Magnetic resonance imaging demonstrated a mass measuring 4 × 5 cm that was suspected to be a hematoma compressing the brachial plexus in a space between the subscapular muscle and the pectoralis minor muscle. Surgery was performed on the third day after injury. In intraoperative observations, the axillary artery was occluded with thrombus along 5 cm; a subscapular artery was ruptured; the brachial plexus was compressed by the hematoma. After evacuation of the hematoma, neurolysis of the brachial plexus, and revascularization of the axillary artery, the patient had an excellent functional recovery of the affected upper limb, postoperatively.

**Conclusion:**

Surgeons should be aware that axillary artery injuries may even occur in young people after severe blunt trauma in the shoulder region and can be associated with secondary brachial plexus injury due to a hematoma. For treatment in cases with progressive nervous deficit after trauma, not only reconstruction of the injured artery but also immediate evacuation of the hematoma, and exploration of the brachial plexus are necessary to avoid irreversible neurological damage.

## Introduction

Neurovascular injury in the shoulder region following blunt trauma that is not associated with either anterior dislocation of the shoulder or fracture of the humeral neck has been rarely reported [1-6]. Furthermore, revision of the literature showed that there are only a few case reports of delayed onset of brachial plexus palsy due to hematoma or pseudoaneurysm formation following trauma in the shoulder region [7-9]. However, in these reports, this condition only affected old patients who had sclerotic, non-elastic vessels. Here, we report a young patient who suffered axillary artery injury combined with delayed brachial plexus palsy that occurred tardily due to compressive hematoma after blunt trauma to the shoulder. The patient was successfully treated using surgical intervention.

## Case presentation

**Figure 1:**
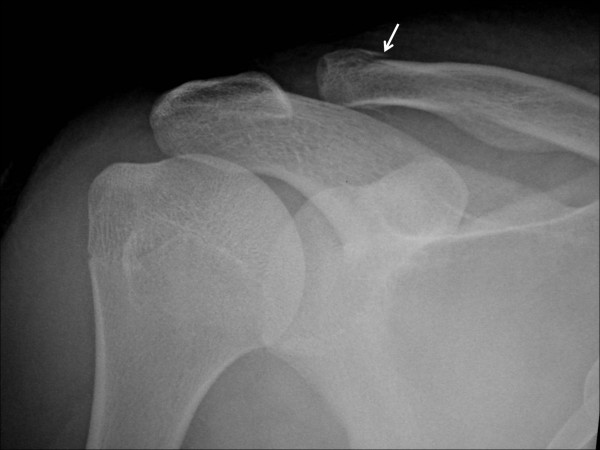
**Roentgenogram at the time of injury**. A non-displaced fracture (white arrow) at the distal end of the right clavicle was observed.

**Figure 2:**
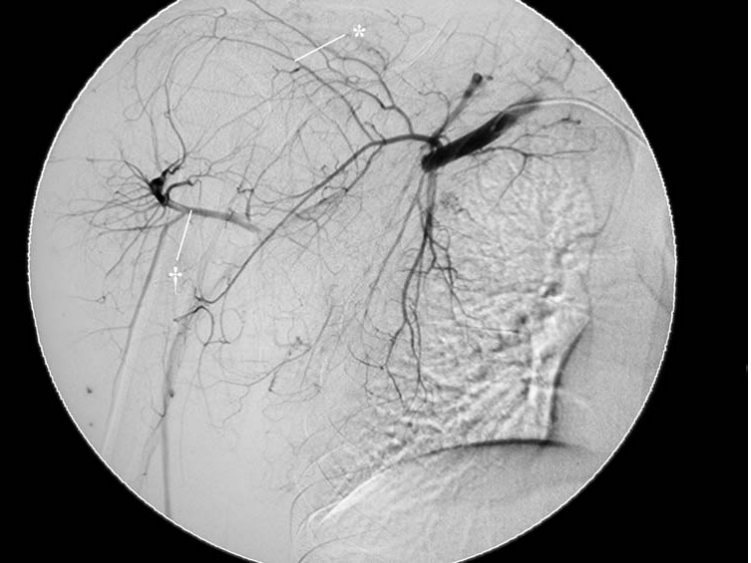
**Selective digital subtraction angiography of the axillary artery**. The axillary artery was completely occluded at the site of the coracoid process of the scapula, and collateral flow through the thoracoacromial (*) and the posterior circumflex humeral (†) arteries existed.

**Figure 3:**
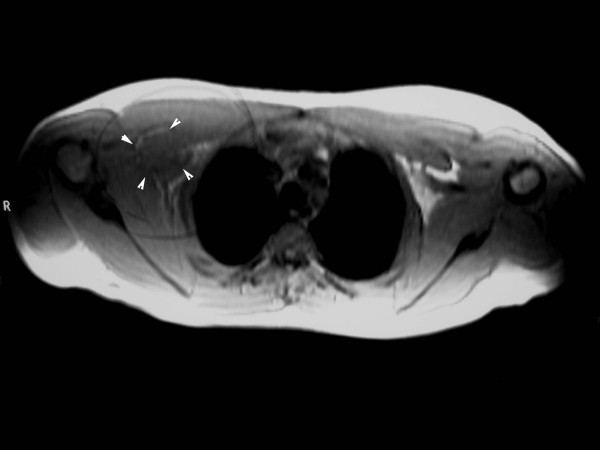
**T1-weighted magnetic resonance imaging scan**. A large mass (white arrowheads) that was suspected to be a hematoma was present, compressing the brachial plexus in a space between the subscapular and pectoralis minor muscles.

**Figure 4:**
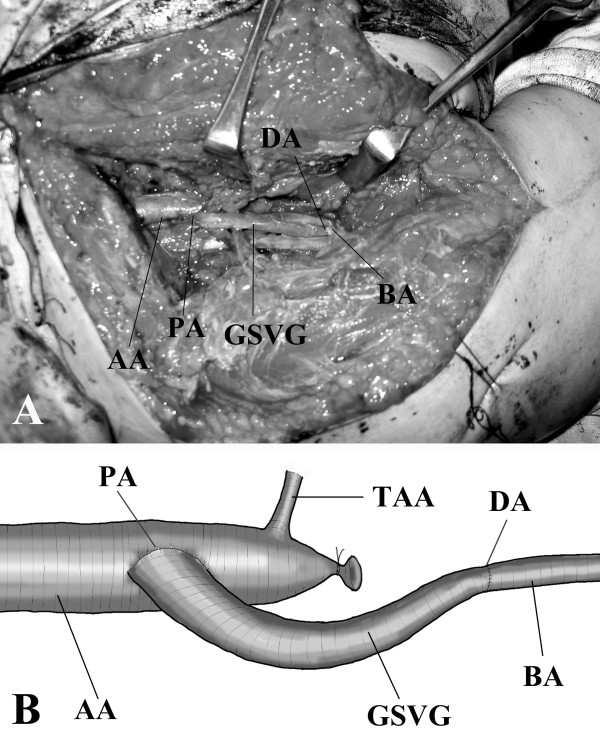
**Intraoperative image of revascularized site**. A: Photograph, B: Scheme. Revascularization of the injured axillary artery was achieved using an interpositional great saphenous vein graft. The proximal and distal sites of the graft were anastomosed using side-to-end and end-to-end methods, respectively. AA: Axillary artery, PA: Proximal anastomosis, GSVG: Great saphenous vein graft, DA: Distal anastomosis, BA Brachial artery.

**Figure 5:**
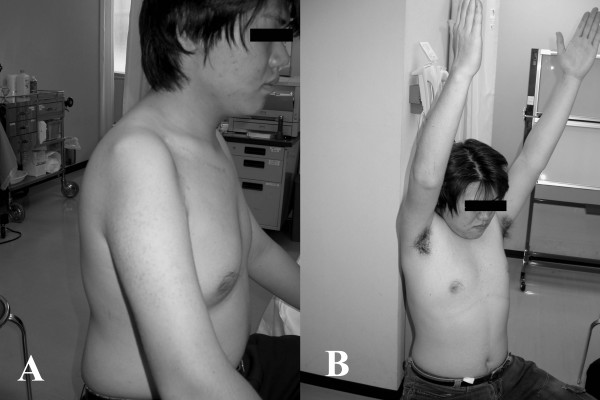
**Analysis of the function of the affected upper arm one year after surgery**. A: Atrophy of the deltoid muscle was observed. B: The patient could abduct his shoulder, and did not have any significant inconvenience with his right arm, in his daily life.

A 16-year-old male who injured his right shoulder in a motorbike accident presented himself to a regional general hospital on the same day of the injury. On initial physical examination, he complained of tenderness of the shoulder and axillary regions. Roentgenograms revealed a non-displaced fracture at the distal end of the right clavicle but neither dislocation of the glenohumeral joint nor humeral neck fracture was observed (Figure 1). Blood supply to the right arm was intact, and the pulses of the radial and ulnar arteries in the affected right arm were palpable. Blood pressure difference between the right and left upper arms was not evaluated at that point. Neurological examination revealed sensory deficit on the lateral aspect of the right upper arm. Although the patient could elevate the upper arm more than 90° against gravity, muscle contraction of the deltoid muscle was not seen under voluntary elevation of the upper arm. There was no other sensory or muscle power deficit. At that point, axillary nerve palsy was diagnosed, and an electrophysiological examination was scheduled for the detailed evaluation of other nerves. However, on the third day after the accident, the patient developed swelling and tenderness in his right upper arm and shoulder, and became paralyzed in his right arm. He was referred to our department. On physical examination, the pulses of the radial and ulnar arteries of the right arm were significantly diminished compared to those in the left arm. Blood pressure was 42/20 mm Hg (systolic/diastolic) in the right arm, and 122/70 mm Hg in the left arm. Widespread ecchymoses were observed on the medial aspect of the upper arm. On neurological examination, muscle power in the right arm included brachialis (M0), biceps (M0), triceps (M0), pectoralis major (M3), pronator teres (M3), supinator (M1), flexor carpi radialis (M3), flexor carpi ulnaris (M3), extensor carpi radialis longus/brevis (M2), flexor digitorum profundus/sublimis (M4), flexor policis longus (M4), extensor digitorum communis (M0), extensor policis longus (M0), and intrinsic muscles (M2) according to the classical medical research council scale. Muscle power of the deltoid and supra/infra spinatus muscles could not be evaluated exactly due to shoulder pain from the fractured clavicle. However, the patient could elevate the upper arm 120° against gravity, indicating that the suprascapular nerve was intact. Complete sensory loss was observed in the lateral aspect of the upper arm and dorsoradial aspects of the forearm and hand, and the volar aspects of the forearm and hand were slightly numbed. The patient complained of slight dull pain (neurostenalgia) at the radial aspect of the forearm. Selective digital subtraction angiography of the axillary artery through the right femoral artery showed complete occlusion of the axillary artery at the site of the coracoid process of the scapula (Figure 2). Collateral blood circulation to the forearm was preserved through the thoracoacromial and posterior circumflex humeral arteries. Magnetic resonance imaging (MRI) demonstrated a mass measuring 4 × 5 cm that was suspected to be a hematoma compressing the axillary artery and brachial plexus in a space between the subscapular muscle and the pectoralis minor muscle (Figure 3A and 3B). On the same day of referral, surgical explorations of the axillary artery and brachial plexus, and evacuation of the hematoma were performed under general anesthesia. At intraoperative observations, the axillary artery was found to be occluded with thrombus along 5 cm, at the site where the pectoralis minor muscle overlaid the artery; however, the adventitia of the artery was not ruptured. A subscapular artery was ruptured at the site of origin from the axillary artery; the brachial plexus was compressed by the hematoma along a dorsal direction. Following evacuation of the hematoma, the infraclavicular part of the brachial plexus was explored from the origin of the cords to the terminal branches. Macroscopically, the brachial plexus was intact. Intraoperative electrostimulation on the terminal branches of the infraclavicular brachial plexus was performed. Although stimulation of the nerves other than the axillary nerve induced the contraction of the innervated muscles, deltoid muscle contraction didn't occur after the stimulation of the axillary nerve. After exploration of the axillary nerve, it appeared continuous, but tension was slackened. External neurolysis of the nerve was performed to promote spontaneous recovery. The axillary artery was transected to observe the interluminal conditions. The artery was occluded by thrombus; the intima was damaged. The intraluminal thrombus was removed using a 4 French Fogarty catheter in both proximal and distal directions, and revascularization was established by performing an interpositional great saphenous vein graft. The proximal and distal sites of the graft were anastomosed using side-to-end and end-to-end methods, respectively. Care was taken not to sacrifice collateral blood circulation (Figures 4A and 4B). The ruptured subscapular artery was not repaired but ligated because the artery was stretched and damaged. Heparin and regional thrombolytic therapy with urokinase were administered intravenously to the patient for 5 days after surgery as an anticoagulatory treatment. Just after the surgery, both the radial and ulnar arteries of the right arm became pulsatile, as in the left arm. The sensorimotor functions started to recover from the first day after surgery. All nervous functions except for that of the axillary nerve were sufficiently recovered within 3 days. One year after surgery, muscular powers except for that of the deltoid muscle were completely recovered. Despite of the atrophy of the deltoid muscle, muscle contraction was seen under voluntary elevation of the upper arm against gravity. The patient could abduct his shoulder 180° with a 10-kg load by conjoint function with the supra spinatus muscle. Electromyographic examination of the deltoid muscle revealed motor units under voluntary control, mild decrease in interference patterns and re-innervations, confirming the partial recovery of muscle function. Sensory disturbance on the lateral aspect of the right upper arm also recovered to normal sensation. The patient was satisfied with the function of his right arm (Figures 5A and 5B).

## Discussion

Cases of delayed brachial plexus palsy due to a hematoma from a ruptured subscapular artery without association of either shoulder dislocations or humeral neck fractures have been previously reported [1,6]. In previous reports, the affected patients were elderly people with non-elastic vessels, and the etiology was advanced arteriosclerosis that can be vulnerable to stretching force in shoulder trauma. However, our present case was only 16 years old. Injuries of the axillary artery and/or its branches can affect young patients who have suffered blunt injuries in the shoulder region even if fractures and/or joint dislocation were not associated.

Axillary artery injury might be accompanied with brachial plexus injury with an incidence rate of 27–44% [10]. The usual mechanisms of brachial plexus injuries involve direct impact or forceful stretch with extreme movement of the neck and/or arm; and symptoms usually occur just after the injury. In the present case, nervous deficits except for those from the axillary nerve injury occurred tardily from the third day after the initial injury. We postulated that the mechanism of nerve injuries in the present case occurred as follows. A hematoma from the ruptured subscapular artery formed, and nerve compression by the hematoma and/or swollen soft tissues promoted nerve palsy. The development of the hematoma was suspected to have led to gradual progressive brachial palsy. Ischemia of the nerves due to axillary artery occlusion might play a subsidiary role in causing brachial palsy. This possible cause of neuropathy after shoulder injury has been reported by Stenning et al. [11]. The intraoperative findings showed that the hematoma was located dorsally to the cords of the brachial plexus; the cords were compressed by the hematoma against the swollen pectralis minor muscle. In the present case, nervous disturbance was more significant in the muscles and sensory territory that were innervated by the posterior cord than the lateral or medial cords. These findings could be supported by the fact that the posterior cord was most closely located to the hematoma; and the posterior cord could be directly compressed by the hematoma. Additionally, in the triangular space formed by the subscapularis muscle, pectoralis minor muscle, and thorax where the cords are located, the medial part of this space had more space than on the lateral part. This might explain the fact that the muscle and sensory territories that were innervated by the medial cord were the least impaired.

According to the critical analysis and intraoperative findings of the present case, the etiology of axillary nerve palsy was more likely the direct stretching of the nerve at the time of injury rather than compression by a posttraumatic hematoma. Hyperabduction of the glenohumeral joint resulted in stretch injury of the axillary nerve. Intraoperative findings of the loosened but continuous axillary nerve supported this speculation. The severity of the injury of the axillary nerve was categorized as Sunderland's grade 3, in which axon continuity is disrupted by the loss of endoneural tubes but the perineurium is preserved. We performed external neurolysis only to promote spontaneous recovery. Reported prognosis and management programs for axillary nerve palsy have been inconsistent. Although Sunderland [12] stated that most cases of axillary nerve palsy recover spontaneously, Berry et al. [13] described that axillary nerve palsy following blunt trauma without an associated fracture or dislocation tend to result in poor recovery. In their report, only 2 of 8 patients with initial complete axillary nerve palsy following blunt trauma without associated fracture or dislocation recovered well. In the present patient, however, without additional surgery, the function of the deltoid muscle was recovered considerably. The patient did not wish an additional surgery because he did not feel any significant inconvenience in his daily activities. If his shoulder elevation function had been seriously impaired, we would have scheduled nerve grafting with the sural nerve or neurotization of the axillary nerve using the motor branch to the long head of the triceps muscle as a secondary surgery.

## Conclusion

We reported a young male case of axillary artery injury combined with delayed brachial plexus palsy that occurred tardily due to compressive hematoma after blunt trauma to the shoulder. The patient was saved from severe complications by early diagnosis and immediate surgery. Surgeons should thoroughly consider injuries of axillary arteries and/or their branches when dealing with patients with blunt trauma to the shoulder, even in cases with normal or subtle X-ray findings and/or preserved distal pulses at initial assessment to avoid overlooking this condition. Additionally, surgeons should be aware that this condition may even occur in young people or can be associated with secondary brachial plexus injury due to a hematoma. For treatment, not only reconstruction of the injured axillary artery but also immediate exploration of the brachial plexus is required, especially in cases with progressive nervous deficits after trauma to avoid irreversible neurological damage.

## Authors' contributions

KM was responsible to this patient for the whole treatment and performed the surgery as a main surgeon. MM and AY assisted KM in the surgery. HY and KO supervised the surgery and gave several suggestions to complete this manuscript. All authors read and approved the final manuscript.

## Consent

The patient and his family were informed that data obtained would be submitted for publication, and written consent was obtained from the patient and their relatives.
